# Prevalence and Antimicrobial Susceptibility of *Campylobacter* Species with Particular Focus on the Growth Promoting, Immunostimulant and Anti-*Campylobacter jejuni* Activities of Eugenol and Trans-Cinnamaldehyde Mixture in Broiler Chickens

**DOI:** 10.3390/ani12070905

**Published:** 2022-04-01

**Authors:** Ahmed Aljazzar, Marwa I. Abd El-Hamid, Rania M. S. El-Malt, Waleed Rizk El-Gharreb, Sherief M. Abdel-Raheem, Abdelazim M. Ibrahim, Adel M. Abdelaziz, Doaa Ibrahim

**Affiliations:** 1 Department of Pathology, College of Veterinary Medicine, King Faisal University, P.O. Box 400, Hofuf 31982, Saudi Arabia; ajazzar@kfu.edu.sa (A.A.); amibrahim@kfu.edu.sa (A.M.I.); 2 Department of Microbiology, Faculty of Veterinary Medicine, Zagazig University, Zagazig 44519, Egypt; mero_micro2006@yahoo.com; 3 Department of Bacteriology, Zagazig Branch, Animal Health Research Institute, Agriculture Research Center, Zagazig 44516, Egypt; raniaelmalt@yahoo.com; 4 Department of Public Health, College of Veterinary Medicine, King Faisal University, P.O. Box 400, Hofuf 31982, Saudi Arabia; sdib@kfu.edu.sa; 5 Food Control Department, Faculty of Veterinary Medicine, Zagazig University, Zagazig 44519, Egypt; 6 Department of Animal Nutrition and Clinical Nutrition, Faculty of Veterinary Medicine, Assiut University, Assiut 71515, Egypt; 7 Department of Pathology, College of Veterinary Medicine, Suez Canal University, Ismailia 41522, Egypt; 8 Faculty of Veterinary Medicine, Zagazig University, Veterinary Educational Hospital, Zagazig 44511, Egypt; adelmmosa@zu.edu.eg; 9 Department of Avian Diseases, El-Ahsaa Veterinary Laboratory, Ministry of Environment, Water and Agriculture, P.O. Box 400, El-Ahsaa 31982, Saudi Arabia; 10 Department of Nutrition and Clinical Nutrition, Faculty of Veterinary Medicine, Zagazig University, Zagazig 44519, Egypt; doibrahim@vet.zu.edu.eg

**Keywords:** eugenol, trans-cinnamaldehyde, XDR, campylobacter, growth performance, broiler chickens, cytokines

## Abstract

**Simple Summary:**

*Campylobacter* species are the leading cause of foodborne bacterial enteritis worldwide. Recently, extensively drug-resistant (XDR) and multi-drug-resistant (MDR) *Campylobacter* spp. have caused several global crises. Therefore, the present work aims to detect the prevalence and antimicrobial resistance patterns of *Campylobacter* spp. from various chicken sources in Egypt, and to investigate the efficacy of a mixture of eugenol and trans-cinnamaldehyde on the performance and immunity of challenged broilers and also to assess their effects on *C. jejuni* load and virulence gene expression in an in vivo model. Our results showed a high prevalence of campylobacter isolates (67.3%). Of note, 25.7 and 74.3% of campylobacter isolates were XDR and MDR, respectively. Interestingly, a mixture of eugenol and trans-cinnamaldehyde had significant enhancing and antimicrobial effects through improving the growth-performance variables, minimizing the *C. jejuni* fecal loads, and decreasing the *C. jejuni* virulence genes (*flaA*, *virB11*, and *wlaN*) expressions in broilers challenged with *C. jejuni*. Moreover, the mixture of eugenol and the trans-cinnamaldehyde had immunostimulant and anti-inflammatory activities. In conclusion, our findings suggest that the utilization of the mixture of eugenol and trans-cinnamaldehyde has a growth-promoting role and can be considered as a better replacement of the antimicrobial agents for the control and treatment of campylobacter infection in broiler chickens.

**Abstract:**

*Campylobacter* species (spp.) are one of the most important causes of human bacterial gastroenteritis in foods of animal origin. Recently, with the spread of multi-drug-resistant (MDR) and extensively drug-resistant (XDR) *Campylobacter* spp., natural alternative therapeutic methods are urgently required. Phytogenic active principles have gained considerable attention due to their proficiency to enhance gut health and, thereby, performance of broiler chickens. Thus, the current study aims to determine the prevalence and antimicrobial resistance of *Campylobacter* spp. of different chicken sources in Sharkia Governorate, Egypt, and to assess the growth-promoting, immunostimulant and antimicrobial effects of a mixture of eugenol and trans-cinnamaldehyde in an in vivo approach. A total of 101 (67.3%) campylobacter isolates was identified, according to both phenotypic and genotypic techniques. Moreover, all of the campylobacter isolates were resistant to erythromycin, trimethoprim/sulfamethoxazole, and ampicillin (100% each). Of note, a dietary supplementation of the mixture of eugenol and trans-cinnamaldehyde led to a significant improvement of the feed conversion ratio and body weight gain and a decrease in the cecal *C. jejuni* loads in the broilers challenged with XDR *C. jejuni*. Additionally, eugenol and the trans-cinnamaldehyde mixture had protective activities via the down-regulation of XDR *C. jejuni* (*flaA*, *virB11* and *wlaN*) virulence genes and proinflammatory cytokines (*TNF-α*, *IL*-2, *IL-6*, and *IL-8*), and the up-regulation of anti-inflammatory cytokine *IL-10*. Thus, we recommend the usage of a mixture of eugenol and trans-cinnamaldehyde as an alternative to antimicrobials for the control and treatment of campylobacter infections.

## 1. Introduction

For more than a century, there has been an evolution in the knowledge about the public health importance of human campylobacter infection. Campylobacteriosis is a global foodborne bacterial gastrointestinal infection and the majority of human campylobacter infection cases are caused by *Campylobacter jejuni* (*C. jejuni*) and, to a lesser extent, *Campylobacter coli* (*C. coli*) [1]. Since 2005, *Campylobacter* species (spp.) have been recognized as the most common cause of human foodborne enteritis in the European Union [2,3]. Furthermore, campylobacter infection is hyperendemic, mainly in young children and infants, in developing countries [4,5]. *Campylobacter* spp. are ubiquitous microorganisms and they are commensal bacteria in the gastrointestinal tract of poultry and domestic animals. Human campylobacteriosis cases usually occur by the consumption of contaminated water, raw or uncooked meat, mainly poultry and its products and unpasteurized milk [2,6].

The majority of campylobacter infection cases are self-limiting and antimicrobial therapy is not indicated; however, antimicrobial treatment may be necessary under specific clinical conditions, such as immunocompromised individuals and patients with persistent or severe symptoms, prolonged or intense enteritis, extraintestinal infections, and bacteremia [2,4]. In these specific circumstances, the treatment may be complicated because of the emergence of multi-drug-resistant (MDR) and extensively drug-resistant (XDR) *Campylobacter* spp., due to the excessive uncontrolled utilization of antimicrobials in the poultry industry, livestock breeding, and veterinary medicine [7,8]. The drugs of choice in the treatment of human campylobacter infections are erythromycin (macrolides) and ciprofloxacin (fluoroquinolones (FQs)) [9]. Recently, *Campylobacter* spp. has become resistant to multiple antimicrobial classes, such as macrolides, FQs, aminoglycosides, tetracyclines, and beta-lactams, which result in increasing the infections with MDR and XDR *Campylobacter* spp. [10].

For public health significance, it is important to identify the pathogenicity markers in campylobacter isolates, which are detected in food. *Campylobacter* spp. have several virulence factors, such as adhesions, acid resistance, heat and cold stress resistance [11], toxin production, hemolysin, capsule [12] and the flagella, and its secreted proteins [13]. The Flagellin A (*flaA*) gene is an important virulence marker in *Campylobacter* spp., which is responsible for the flagella formation and, consequently, bacterial adhesion, invasion, and motility [14]. Additionally, *virB11*, a plasmid-encoded gene, is correlated to the host cell invasion [5] and the *wlaN* gene is associated with the production of lipo-oligosaccharide and a β-1,3 galactosyltransferase. The *wlaN* gene products imitate the myelin sheath ganglioside structure on nerve cells, which result in the development of Guillain–Barré syndrome, an acute peripheral polyneuropathy, after campylobacter infection [15]. 

Chickens have been identified as the main reservoir of *Campylobacter* spp., and it is responsible for up to 80% of human campylobacteriosis cases. Human infection usually occurs through the handling and consumption of chicken meat and its products that are contaminated during the slaughtering and processing of carcasses [4,15]. Therefore, there is an important demand for effective protocols to control *Campylobacter* spp. at the farm level through minimizing the prevalence of *Campylobacter* spp. in poultry products, which will result in reducing the contamination of products and the prevalence of campylobacteriosis in humans [16]. Additionally, reducing campylobacter colonization, adhesion, and invasion of the intestinal epithelial cells through minimizing the production of their virulence genes and enhancing the immune response, might control the campylobacteriosis in humans [17].

Recent studies reported the capability of natural antimicrobials in controlling *Campylobacter* spp. in chickens, due to the consumers’ increasing demands for safe and natural products that use the least preservatives [18]. Herbal plant extracts, such as phytochemicals, have generally been used since ancient times as food flavoring agents, dietary supplements, and food preservatives to avoid the spoilage of food and for public health improvement. Furthermore, phytochemicals, such as essential oils (EOs), have antimicrobial characteristics [1,17] and are capable of modifying the pro- and anti-inflammatory cytokines and virulence genes’ expressions [19,20]; thus, they are used in herbal medicine for the treatment of various diseases [17]. Additionally, EOs can be utilized in poultry nutrition as feed additives because they enhance poultry growth performance, feed efficiency parameters, and meat quality by improving digestibility [19,20]. Eugenol is a polyphenol complex, which is considered as the primary antimicrobial active ingredient found in clove (*Syzgium aromaticum*) essential oil, whereas trans-cinnamaldehyde, an aldehyde found in the bark of the cinnamon (*Cinnamomum zeylandicum*) tree, is the principal component of cinnamon EO. The aforementioned EOs are classified by the Food and Drug Administration (FDA) under “Everything Added to Food in the United States” and as Generally Recognized as Safe (GRAS) with fast biodegradation and least cytotoxicity, and they can be used in food as good substitutes for the antimicrobial agents [17,21].

As the problem of resistant foodborne bacteria rises, infection with MDR pathogens will be substituted by XDR strains. Thus, we predict the worldwide spread of XDR foodborne bacteria, especially *Campylobacter* spp., soon. Eugenol and trans-cinnamaldehyde were reported as effective therapeutic options; however, to our best knowledge, no reports evaluated their activity against multi-virulent XDR *Campylobacter* spp. in vivo. Moreover, when a blend is utilized, the active principles might have synergistic effects influencing their modes of actions [22]. Hence, the present study aims to (i) detect the prevalence and antimicrobial resistance patterns of *Campylobacter* spp. from various chicken sources in Egypt; (ii) determine the virulence profiles of XDR campylobacter isolates; and (iii) assess, for the first time, the in vivo efficacy of a mixture of eugenol and trans-cinnamaldehyde, in comparison to the most susceptible examined antibiotics on the growth performance and expression of cytokines-related genes in broiler chickens experimentally infected with XDR and multi-virulent *C. jejuni*, in addition to investigating their activities on the viability and expression of the virulence genes of the infecting *C. jejuni* strain.

## 2. Materials and Methods

### 2.1. Ethical Statement

The experimental protocols were conducted following the regulations and approved guidelines of the Institutional Animal Care and Use Committee of Faculty of Veterinary Medicine, Zagazig University, Egypt under the reference number of ZU-IACUC/2/F/263/2021.

### 2.2. Sample Collection

A total of 150 different samples were collected from recently slaughtered broiler chickens (Ross 308) of 6 weeks of age during the period ranging from August 2019 to October 2020 (14 months) from different areas in Zagazig city, Sharkia Governorate, Egypt. A total of 11 samples were obtained per month from the 11 main chicken processing plants in Zagazig city, Egypt (*n* = 11, 14 samples from each outlet), including chicken luncheon meats, liver, breast meats, cecal parts, and cloacal swabs (*n* = 30 each), and 1 site was randomly tested per bird. Each sample represented a single bird (the samples were completely independent and randomly picked). The cloacal swabs and 25 g of each sample were transported directly into 225 mL of supplemented Bolton broth (Oxoid, Cambridge, UK), leaving a headspace of about 20 mm in the tightly capped tubes to generate microaerophilic conditions [23]. The obtained samples were aseptically transferred into an icebox and to the laboratory as soon as possible for the isolation and identification of *Campylobacter* spp.

### 2.3. Isolation and Identification of Campylobacter Species

For *Campylobacter* spp. isolation, the obtained specimen in Bolton broth was incubated at 42 °C/24–48 h in darkness in a microaerophilic atmosphere (85% N_2_, 10% CO_2_, and 5% O_2_) utilizing an anaerobic jar (Sigma-Aldrich, St. Louis, MO, USA) and CampyGen sachets (Oxoid, Cambridge, UK). Subsequently, 0.1 mL of the inoculated Bolton broth was inoculated onto the surface of supplemented modified charcoal cefoperazone deoxycholate agar (mCCDA) plates (Oxoid, Cambridge, UK), then the inoculated plates were incubated at 42 °C/48 h in a microaerophilic atmosphere. For further purification, the suspected campylobacter colonies were cultivated on 5% sheep blood agar plates (Oxoid, Cambridge, UK), then incubated at 42 °C/48 h in a microaerophilic atmosphere. Next, the suspected colonies were presumably identified through their culture characters on mCCDA and blood agar, Gram’s staining, motility test, susceptibility to nalidixic acid and cephalothin, and biochemical identification procedures, including oxidase, catalase, rapid sodium hippurate, and indoxyl acetate hydrolysis [23].

### 2.4. Antimicrobial Susceptibility Testing

The standard Kirby–Bauer disc diffusion method [24] was utilized to test the susceptibility of all the recovered campylobacter isolates to 18 antimicrobials. Briefly, a few (3–10) colonies from a single sample were suspended in sterile physiological saline and compared to the 0.5 McFarland standard solution. Then, Mueller–Hinton agar (Oxoid, Cambridge, UK) plates contained 5% of defibrinated sheep blood were cultivated with the prepared suspension. Next, the antimicrobial discs were placed onto the surface of dried plates and incubated at 42 °C/48 h in a microaerophilic atmosphere. A total of 18 antimicrobial discs (Oxoid, UK) that fall into 10 various antimicrobial categories, which were usually utilized in veterinary and human medicine in Egypt, were used: chloramphenicol (C, 30 µg); clindamycin (DA, 2 µg); colistin (CT, 10 µg); linezolid (LNZ, 30 µg); tobramycin (TOB, 10 µg); gentamicin (CN, 10 µg); amikacin (AK, 30 µg); azithromycin (AZM, 30 µg); erythromycin (E, 15 µg); doxycycline (DO, 30 µg); trimethoprim-sulfamethoxazole (SXT, 1.25 + 23.75µg); nalidixic acid (NA, 30 µg); aztreonam (ATM, 30 µg); imipenem (IMP, 10 µg); cefoxitin (FOX, 30 µg); sulbactam-ampicillin (SAM, 10 + 10 µg); ampicillin (AM, 10 µg); and ciprofloxacin (CIP, 5 µg). The susceptibility of the examined isolates was measured via the determination of the diameter of the inhibition zone of the antimicrobial discs, and the results were explained following the guidelines of the Clinical and Laboratory Standards Institute (CLSI) to classify each antimicrobial agent as either susceptible, intermediate, or resistant [25]. The XDR was defined as the non-susceptibility of an isolate to all antimicrobial agents, except two or fewer antimicrobial classes; meanwhile, MDR was described as the non-susceptibility of campylobacter isolates to at least one antimicrobial agent in three or more unrelated antimicrobial classes [26]. The multiple antibiotic resistance (MAR) indices were determined for the campylobacter isolates through the following equation: MAR = a/b, where (a) represents the number of antimicrobials to which the examined isolates were resistant and (b) is the total number of antimicrobial agents utilized [27].

### 2.5. Conventional PCR Assay

The PCR assays conducted in the current work were carried out on the highly resistant campylobacter isolates. The DNA extraction was performed utilizing the QIAamp DNA Mini Kit (Qiagen, Germantown, MD, USA), following the manufacturer’s instructions. Conventional PCR amplification procedures were carried out for the detection of the *23S rRNA*, *ceuE*, and *mapA* genes of genus *Campylobacter*, *C. coli*, and *C. jejuni*, respectively. Additionally, three significant virulence genes (*wla*N, *vir*B11, and *fla*A) were also detected via PCR assays. All PCR protocols were performed, in triplicate, using the Emerald Amp GT PCR Master Mix (Takara, Mountain View, CA, USA), following the instructions of the manufacturer. The primer sequences for the tested genes in all the PCR procedures are presented in Table 1. All protocols of the PCR amplification were carried out as previously described [28,29,30]. Subsequently, ethidium bromide staining (Sigma-Aldrich, St. Louis, MO, USA) and agarose gel electrophoresis for the PCR products’ visualization were performed [5,31]. In all the PCR procedures, negative controls (no template DNA) were PCR grade water and positive controls were the reference strains of *C. coli* (NCTC11366) and *C. jejuni* (NCTC11322).

### 2.6. In Vivo Assessment of the Efficacy of the Eugenol and Trans-Cinnamaldehyde Mixture

One XDR and multi-virulent *C. jejuni* strain was selected for a further challenge experiment to assess the efficacy of the mixture of eugenol and trans-cinnamaldehyde, in comparison to the most effective examined antibiotic on the growth performance and the expression of the cytokines-related genes of broilers, and to also investigate their activities on the viability and expression of the virulence genes of the infecting strain.

#### 2.6.1. Plant-Derived Antimicrobials

The eugenol and trans-cinnamaldehyde used in the current experiment were purchased from Sigma-Aldrich (St. Louis, MO, USA). Eugenol is a polyphenol complex that is considered as the primary antimicrobial compound found in clove EO, whereas trans-cinnamaldehyde is an aldehyde found in the bark of the cinnamon tree and it is the main component of cinnamon EO.

#### 2.6.2. Experimental Infection by XDR and the Multi-Virulent *C. jejuni* Strain

An XDR and multi-virulent *C. jejuni* strain were used in the current experimental trial, in which the challenge was conducted orally at 23 days of age via the crop gavage with 1 mL of 10^8^ CFU/mL of the bacterial inoculum. The bacterial infection was checked via the re-isolation and identification of the infecting strain. Additionally, the re-examination of its antimicrobial resistance and virulence genes profiles were carried out to make sure that the isolated strain corresponded to the infecting one.

#### 2.6.3. Experimental Broiler Chickens, Design, and Feeding Regime

This experimental trial was conducted on 400 1-day-old broiler chicks (Ross 308) that were purchased from a local commercial poultry hatchery. On arrival, the chicks were weighed separately and randomly assigned into 4 groups in floor pens (100 birds in each pen, with 5 replicates/group and 20 chicks/replicate). The chicks in the negative control (NC) group were kept unchallenged, while the chicks in the positive control (PC) group were challenged. The chicks in the NC and PC groups were fed the basal diet without any supplementation. The PC and the other 2 treatment groups were challenged with XDR and a multi-virulent *C. jejuni* strain at 23 days of age. The EOs treatment group was fed a basal diet supplemented with a mixture of eugenol and trans-cinnamaldehyde (1:1) at a concentration of 400 mg/kg each from the first day of life as a prophylactic. Moreover, in the antibiotic treated group, all the birds were treated intramuscularly with 50 mg/kg of cefoxitin after the appearance of clinical signs (lethargy, depression, and decreased body weight) and re-isolation of the bacterium 3 days post infection. All chicks were kept under completely hygienic environments, according to the Ross Broiler Management Guide [33]. All the chicks were permitted free access to feed and drinking water throughout the 37-day experimental period. All the birds were fed coccidiostat- and antibiotic-free diets in the mash form for the starter (days 1–10), grower (days 11–20), and finisher (days 21–37) periods, according to the Ross broiler nutrition specifications [33], as shown in Table 2. All the feed constituents and diets were examined chemically for the determination of crude fiber, crude protein, moisture, and ether extract, and these tests were in accordance with the Association of Official Analytical Chemists [34].

#### 2.6.4. Monitoring the Growth Performance of the Broilers

The body weight (BW) and feed intake (FI) were recorded for calculating the cumulative body weight gain (BWG) and feed conversion ratio (FCR) over the entire experimental period (1–37 days), as previously described [35,36,37,38].

#### 2.6.5. Sampling

A total of 5 chicks from each replicate were slaughtered and sacrificed, and the cecal contents from each chick were aseptically removed and kept frozen at −80 °C in sterile tubes for the re-isolation and identification of the infecting *C. jejuni* strain and further quantification of the campylobacter populations through a quantitative real-time PCR (qPCR) assay and the analysis of the mRNA expression of campylobacter virulence genes via the reverse transcription quantitative polymerase chain reaction (RT-qPCR) assay at 30 (7 days post-infection, dpi) and 37 (14 dpi) days of age. Additionally, the spleen was rinsed with sterile phosphate-buffered saline and used for investigating the expression of cytokine-related genes using RT-qPCR assay 7 and 14 dpi.

#### 2.6.6. Gene Expression Analysis by the Reverse Transcription Quantitative PCR Assay

Splenic tissues were utilized for detecting the mRNA expression levels of cytokines-related genes (tumor necrosis factor-alpha (*TNF-α*), interleukin (*IL*)-2, *IL-6*, *IL-8*, and *IL-10*), and the cecal contents were utilized for the subsequent investigation of the expression levels of campylobacter virulence genes (*fla*A, *vir*B11, and *wla*N). The total RNA was extracted utilizing the QIAamp RNeasy Mini kit (Qiagen, Hilden, Germany), according to the instructions of the manufacturer. The concentration of the extracted RNA was determined at 260 nm and the RNA clarity was determined utilizing a Spectrostar NanoDropTM 2000 spectrophotometer (Thermo Fisher, Sunnyvale, CA, USA). A one-step RT-qPCR assay was conducted, in triplicates, utilizing a QuantiTect SYBR Green RT-PCR Kit (Qiagen, Hilden, Germany) via the Strata-gene MX3005P real-time PCR identification system (Thermo Fisher, Sunnyvale, CA, USA), following the guidelines of the manufacturer. A melting curve analysis was used for the verification of all PCR amplifications. The transcript’s expression levels were normalized using glyceraldehyde 3-phosphate dehydrogenase (*GAPDH*) and campylobacter *23S rRNA* genes as endogenous controls. The primer sequences of the virulence and cytokines-related genes used in the RT-qPCR assays are shown in Table 1 and Table 3, respectively. The relative mRNA expression data of the tested genes were assessed using the 2^−∆∆Ct^ method [39].

#### 2.6.7. Quantification of the Campylobacter DNA Copies by Quantitative Real-Time PCR Assay

DNA was extracted from the cecal contents using a QIAamp DNA stool Mini Kit (Qiagen, Hilden, Germany), according to the recommendations of the manufacturer. The absolute quantification of the campylobacter populations was conducted via qPCR assays, in triplicate, utilizing the Stratagene MX3005P RT-PCR machine and QuantiTect SYBR Green PCR Master Mix (Qiagen, Hilden, Germany), following the guidelines of the manufacturer. The primer sequence of the *23S rRNA* gene is shown in Table 1. Ten-fold serial dilutions of the extracted DNA from pure campylobacter cultures were conducted to generate the standard calibration curves for the qPCR. The number of target genomic DNA copies were measured and the campylobacter concentration was expressed as the log_10_ CFU/gram of the cecal content.

### 2.7. Statistical Analysis

The results were examined statistically via the SPSS Inc. software version 26 (IBM Corp., Armonk, NY, USA). The chi-squared test was used to analyze the variations in the incidence of *Campylobacter* spp. from various chicken sources, and to assess the variations in the antimicrobial resistance profiles and the prevalence of virulence genes in the recovered isolates from different sources and between *C. jejuni* and *C. coli* isolates. Additionally, the homogeneity and normality between the experimental groups were conducted using Levene’s and Shapiro–Wilk’s tests, respectively. The differences between the data of the experimental trials were expressed as the standard error of the mean (SEM), and the ANOVA and Tukey’s test were used to assess the significant differences between the mean values. If the *p*-value was less than 0.05, it was considered statistically significant. All graphs were produced utilizing the GraphPad Prism software Version 8 (San Diego, CA, USA).

## 3. Results

### 3.1. Prevalence of the Campylobacter Species in the Broiler Chickens in Sharkia Governorate, Egypt

According to the phenotypic identification results, a total of 101 (67.3%) campylobacter isolates were recovered from 150 various samples obtained from broiler chickens in Sharkia Governorate, Egypt. The prevalence of campylobacter isolates in the recovered specimens is presented in Table 4. *Campylobacter* spp. were highly distributed between chicken cloacal swabs (90%) and cecal parts (86.7%), while chicken luncheon meats were campylobacter negative (Table 4). Furthermore, *C. jejuni* was the most common *Campylobacter* spp. (54%), followed by *C. coli* (13.3%). *Campylobacter jejuni* was highly distributed among chicken cecal parts and cloacal swabs (70% each), followed by breast meats (66.7%). Meanwhile, *C. coli* isolates were more prevalent in the chicken livers and cloacal swabs (20% each), followed by cecal parts (16.7%) (Table 4). Additionally, there were statistically significant variations (*p* ˂ 0.001) in the incidences of *Campylobacter* spp., *C. coli*, and *C. jejuni* between the different sample origins.

### 3.2. Antimicrobial Susceptibility Testing of the Campylobacter Species

All 101 recovered campylobacter isolates were examined for their susceptibility against the examined 18 antimicrobials, as presented in Table 5. Interestingly, the examined campylobacter isolates were completely resistant to trimethoprim/sulfamethoxazole erythromycin and ampicillin (100% each). Additionally, the majority of campylobacter isolates were resistant to clindamycin and nalidixic acid (95% each), followed by azithromycin (91.1%), aztreonam (84.2%), and doxycycline (83.2%). Meanwhile, the lowest resistance rates were detected against cefoxitin (33.7%), amikacin (34.7%), and imipenem (42.6%) (Table 5).

Regarding the isolates’ sources, the antimicrobial resistance patterns of the campylobacter isolates from various chicken sources were different (Table 5). It was observed that all the campylobacter isolates from the chicken liver and breast meat samples were 100% resistant to nalidixic acid, while the campylobacter isolates from the chicken cloacal swabs were 100% resistant to clindamycin. Additionally, statistical significance variations were observed in the resistance patterns between the campylobacter isolates from the different chicken sources to nalidixic acid, aztreonam, and cefoxitin (*p* = 0.03, 0.018, and 0.048, respectively). Additionally, there was a higher statistically significant difference in the linezolid resistance rates among the campylobacter isolates from the different chicken sources (*p* = 0.001) (Table 5). Meanwhile, there were no statistically significant differences (*p* ˃ 0.05) in the resistance percentages among the campylobacter isolates from the different chicken sources to the other examined antimicrobial agents.

**Figure 1:**
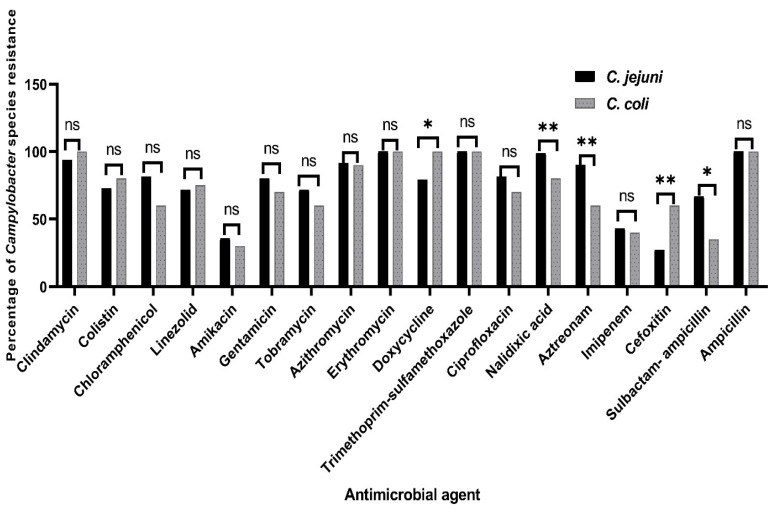
Prevalence of *Campylobacter jejuni* and *C. coli* resistance against 18 antimicrobial agents. ns: non-significant, * *p* < 0.05, ** *p* < 0.01.

According to the species level, our findings present that *C. jejuni* isolates are more resistant than *C. coli* isolates to the tested antimicrobial agents, except for clindamycin, colistin, linezolid, doxycycline, and cefoxitin (Figure 1). Moreover, statistical significance variations were detected in the resistance rates among the *C. coli* and *C. jejuni* isolates to doxycycline and sulbactam-ampicillin (*p* = 0.021 and 0.012, respectively). Additionally, higher statistical significance differences were observed in the resistance rates between the *C. jejuni* and *C. coli* isolates to cefoxitin, aztreonam, and nalidixic acid, (*p* = 0.008, 0.003, and 0.005, respectively). However, no statistical significance variations (*p* ˃ 0.05) were observed in the resistance patterns among the *C. coli* and *C. jejuni* isolates to the other investigated antimicrobials (Figure 1).

**Figure 2:**
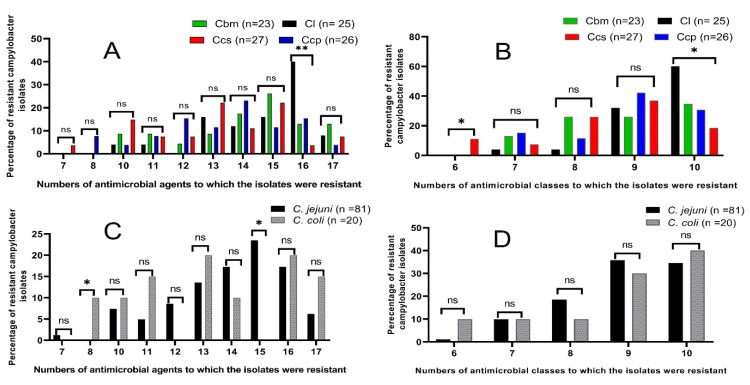
Antimicrobial resistance patterns of the total campylobacter isolates from different chicken sources (**A**,**B**) and *C. jejuni* and *C. coli* (**C**,**D**). Cl: chicken liver; Cbm: chicken breast meats; Ccp: chicken cecal parts; Ccs: chicken cloacal swabs, *n*: number, ns: non-significant, * *p* < 0.05, ** *p* < 0.01.

Interestingly, it was found that the *C. jejuni* isolates were more resistant to 8 and 9 antimicrobial categories (18.5 and 35.8%, respectively) than the *C. coli* isolates (10 and 30%, respectively). Meanwhile, the resistances to 6, 7, and 10 antimicrobial classes were higher in *C. coli* isolates (10, 10, and 40%, respectively) than the *C. jejuni* isolates (1.2%, 9.9%, and 34.6%, respectively). In total, the resistance rates to 10 antimicrobial classes were more prevalent among the campylobacter isolates from the chicken liver samples (60%). Meanwhile, the resistance patterns to 7 and 9 antimicrobials categories were more prevalent among the campylobacter isolates from the chicken cecal parts (15.3 and 42.3%, respectively). Moreover, the resistance proportions to the 8 antimicrobial classes were the highest among the campylobacter isolates from the chicken breast meats (26.1%) and those to the 6 classes were the highest among the campylobacter isolates from the chicken cloacal swabs (11.1%) (Figure 2).

Of note, our results reveal that 26 campylobacter isolates (25.7%) were XDR: 12 (48%), 6 (26.1%), 5 (19.2%), and 3 (11.1%) were obtained from chicken liver, breast meats, cecal parts, and cloacal swabs, respectively; meanwhile, 75 campylobacter isolates (74.3%) were recognized as MDR. Interestingly, our results present that all the tested campylobacter isolates showed an MAR index ≥0.39 (Table 6), which represent high-risk contamination sources, where the antimicrobials are frequently utilized. A total of 8 campylobacter isolates had MAR indices >0.9 (resistance to 17 antimicrobial agents); 2 (8%), 3 (13%), 1 (3.8%), and 2 (7.4%) were isolated from chicken liver, breast meats, cecal parts, and cloacal swabs, respectively (Table 6). Moreover, statistical significance variations were observed in the resistance profiles between the campylobacter isolates from various chicken sources to 16 antimicrobial agents (*p* = 0.006) and to 6 and 10 antimicrobial classes (*p* = 0.037 and 0.017, respectively) (Figure 2A). Additionally, statistical significance variations were observed in the resistance patterns among the *C. jejuni* and *C. coli* isolates to 8 and 15 antimicrobial agents (*p* = 0.038 and 0.024, respectively) (Figure 2B).

### 3.3. Molecular Grouping of the Campylobacter Species from Various Chicken Sources

**Figure 3:**
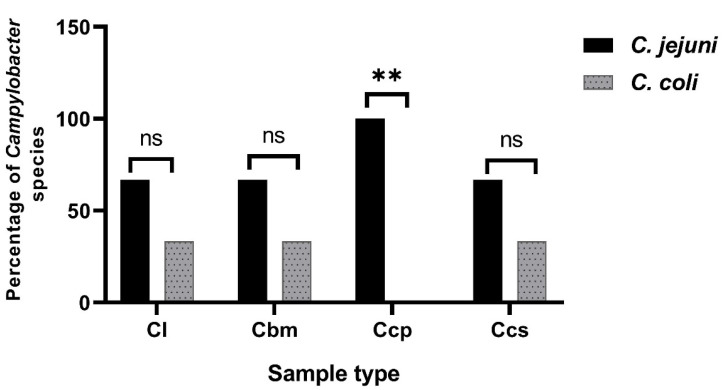
Prevalence of molecularly identified *Campylobacter* species in various chicken samples. Cl: chicken liver; Cbm: chicken breast meats; Ccp: chicken cecal parts; Ccs: chicken cloacal swabs; ns: non-significant; ** *p* < 0.01. The prevalence rate for each species was calculated regarding the total number of the examined samples.

A total of 26 XDR campylobacter isolates (resistant to 16 and 17 antimicrobials) were submitted for conventional PCR assays. Those isolates were recovered from the chicken liver (12), breast meats (6), cecal parts (5), and cloacal swabs (3). All the 26 screened campylobacter isolates (100%) were identified as genus *Campylobacter* by the PCR detection of the *23S rRNA* gene. Additionally, 19 isolates (73.1%) and 7 isolates (26.9%) were positive for *mapA* and *ceuE* genes, respectively, and confirmed to be *C. jejuni* and *C. coli,* respectively. Of the 19 *C. jejuni* isolates, 8 (42.1%), 4 (21.1%), 5 (26.3%), and 2 (10.5%) were recovered from chicken liver, breast meats, cecal parts, and cloacal swabs, respectively. Moreover, 7 *C. coli* isolates were recovered from 4 (57.1%) chicken liver, 2 (28.6%) breast meat, and 1 (14.3%) cloacal swab samples. These findings were consistent with those of the phenotypic identification techniques. Additionally, statistically significant variations (*p* = 0.008) were observed in the incidences of the *C. coli* and *C. jejuni* isolates between the samples of the chicken cecal parts. Meanwhile, no statistical significance differences were observed in the incidence of the *C. coli* and *C. jejuni* isolates between the cloacal swabs, breast meat, and chicken liver samples (*p* = 1, 0.567, and 0.22, respectively) (Figure 3).

### 3.4. Molecular Investigation of the Virulence-Related Genes among the Investigated Isolates

**Figure 4:**
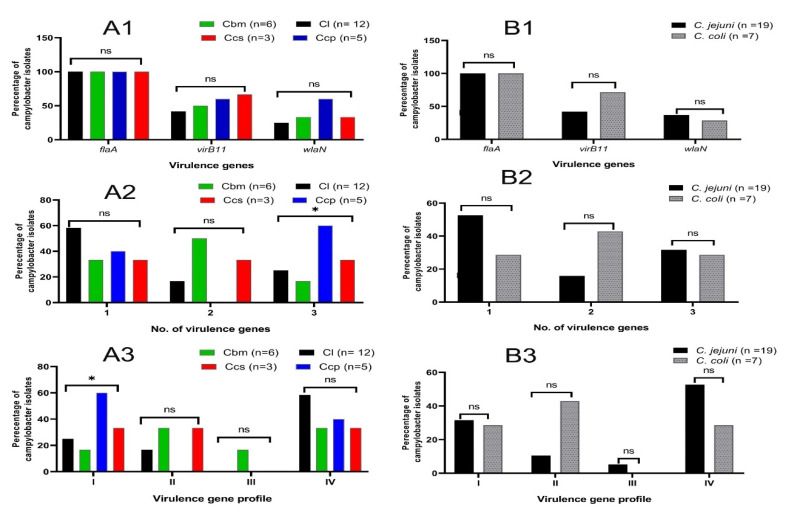
Distribution of the virulence genes among the total campylobacter isolates from the different chicken sources (**A1**–**A3**) and *C. jejuni* and *C. coli* (**B1**–**B3**). Cl: chicken liver; Cbm: chicken breast meats; Ccp: chicken cecal parts; Ccs: chicken cloacal swabs, *n*: number; ns: non-significant; * *p* < 0.05; virulence gene profiles; I: *flaA*, *virB11*, and *wlaN*, II: *flaA* and *virB11*, III: *flaA* and *wlaN*, and IV: *flaA*.

All 26 molecularly confirmed campylobacter isolates were examined for the presence of three significant virulence genes, which have fundamental roles in the pathogenesis of *Campylobacter* spp. (*flaA*, *virB11*, and *wlaN*). Of the 26 examined isolates, 26 (100%) were positive for the *flaA* gene, 13 (50%) were positive for the *virB11* gene, and 9 (34.6%) were positive for the *wlaN* gene. Of note, four virulence gene profiles were detected among the tested campylobacter isolates (Figure 4). A total of 12 XDR campylobacter isolates (46.2%) revealed the most frequent virulence gene profile (*flaA*).

Regarding the isolates’ sources, it was found that the *virB11* gene was more prevalent among the campylobacter isolates from the chicken cloacal swabs (66.7%) and cecal parts (60%). Meanwhile, the *wlaN* gene was more prevalent among the campylobacter isolates from the chicken cecal parts (60%), followed by the cloacal swabs and breast meats (33.3% each). Moreover, 14 (53.8%) campylobacter isolates contained at least 2 virulence genes; 5 (41.7%), 4 (66.7%), 3 (60%), and 2 (66.7%) were recovered from the chicken liver, breast meats, cecal parts, and cloacal swabs, respectively. Additionally, 8 (30.8%) campylobacter isolates harbored 3 investigated virulence genes; 3 (25%), 1 (16.7%), 3 (60%), and 1 (33.3%) were recovered from the chicken liver, breast meats, cecal parts, and cloacal swabs, respectively. There was a statistically significant difference (*p =* 0.048) in the virulence profile I of the campylobacter isolates among the different chicken sources (Figure 4).

According to the spp. level, it was found that the *virB11* gene was more prevalent among the *C. coli* isolates (71.4%), while the *wlaN* gene was more prevalent among the *C. jejuni* isolates (36.8%). Moreover, 5 *C. coli* isolates (71.4%) contained at least 2 virulence genes. Meanwhile, 6 *C. jejuni* isolates (31.6%) harbored 3 investigated virulence genes. Additionally, no statistical significance variations (*p* ˃ 0.05) were observed in the virulence gene profiles between the *C. coli* and *C. jejuni* isolates (Figure 4).

### 3.5. In Vivo Experimental Study

An identified XDR and multi-virulent *C. jejuni* strain, which was resistant to 17 tested antimicrobials, sensitive only to cefoxitin antibiotic and exhibited the virulence profile I (*flaA*, *virB11*, and *wlaN*), was used as a challenge strain in our *in vivo* experiment to assess the growth promoting, immunostimulant, antibacterial, anti-virulence, and activities of the eugenol and trans-cinnamaldehyde mixture.

#### 3.5.1. Growth Performance

The growth performance variables throughout the experimental period are presented in Table 7. Significant differences were observed between the various experimental groups over the entire rearing period. The *C. jejuni* challenge significantly (*p* < 0.05) reduced the BW and BWG and increased the FI and FCR in the PC group, compared to the challenged groups treated with the mixture of eugenol and trans-cinnamaldehyde and cefoxitin. Interestingly, the FCR was most significantly improved (*p* < 0.05) in the NC and the mixture of eugenol and trans-cinnamaldehyde-treated groups, followed by the cefoxitin-treated group, with no significant differences between the NC and the mixture of eugenol and trans-cinnamaldehyde-treated groups. Meanwhile, chicks in the NC, cefoxitin, and the mixture of eugenol and trans-cinnamaldehyde-treated groups exhibited a significant (*p* < 0.05) increased BWG over the entire rearing period, unlike the PC group.

#### 3.5.2. Analysis of Cytokines Genes Expression

**Figure 5:**
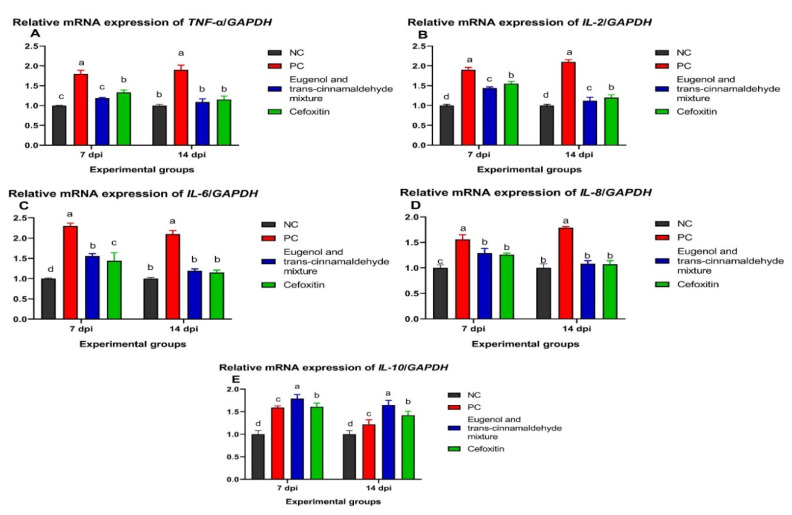
Relative mRNA expression levels of tumor necrosis factor-alpha (*TNF-α*; (**A**)); interleukin-2 (*IL-2*; (**B**)); *IL-6* (**C**), *IL-8* (**D**), and *IL-10* (**E**) in the spleen of chicks supplemented with the mixture of eugenol and trans-cinnamaldehyde and treated with cefoxitin antibiotic at 7 and 14 days post-infection (dpi) with XDR *C. jejuni*. Error bars represent the standard error of the mean (SEM). NC (negative control): chicks fed a basal diet and not challenged; PC (positive control): chicks fed a basal diet and challenged with XDR *C. jejuni*; eugenol and trans-cinnamaldehyde mixture: chicks fed the basal diet supplemented with a mixture of eugenol and trans-cinnamaldehyde; and cefoxitin: chicks fed a basal diet and treated with a cefoxitin antibiotic. All groups, except the NC group, were challenged with XDR *C. jejuni* at 23 days of age. ^a–d^ Means of the columns with different letters indicate a statistically significant difference (*p* < 0.05).

The data of the cytokines genes expression analysis are shown in Figure 5. At 7 and 14 dpi, the relative expression levels of the *TNF-α* gene were significantly decreased (*p* < 0.05) in the mixture of the eugenol and trans-cinnamaldehyde-treated group, followed by the cefoxitin-treated group, in comparison to the PC group, and there was no significant difference between the NC and mixture of the eugenol and trans-cinnamaldehyde-treated groups. Moreover, the most marked reduction in the *IL-2* expression level was observed in the mixture of the eugenol and trans-cinnamaldehyde-treated group, in respect to the PC. Meanwhile, the relative expression levels of the *IL-6* and *IL-8* genes were decreased in the cefoxitin-treated group, followed by the mixture of the eugenol and trans-cinnamaldehyde-treated group. Interestingly, no statistical significance variations were observed among the expression levels of the *IL-6* and *IL-8* genes among the NC, cefoxitin, and eugenol and trans-cinnamaldehyde mixture-treated groups at 14 dpi. Additionally, the *IL-10* gene expression level was significantly upregulated (*p* < 0.05) in the group treated with the mixture of eugenol and trans-cinnamaldehyde, compared to the PC group, at both time points.

#### 3.5.3. Expression Analysis of the *C. jejuni* Virulence Genes via the RT-qPCR Assay

**Figure 6:**
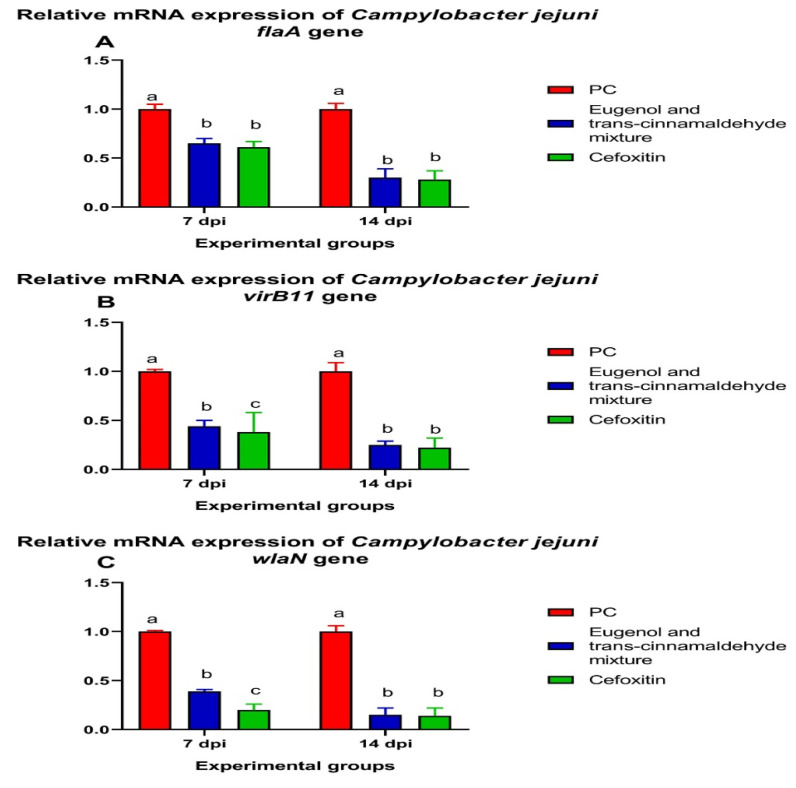
Relative mRNA expression levels of the *C. jejuni flaA* (**A**)*, virB11* (**B**), and *wlaN* (**C**) genes in response to supplementing the mixture of eugenol and trans-cinnamaldehyde and treatment with cefoxitin antibiotic at 7 and 14 days post-infection (dpi) with XDR *C. jejuni*, as were determined via the RT-qPCR assay. The results are expressed as the means ± standard error of the mean (SEM, error bars). PC (positive control): chicks fed a basal diet and challenged with XDR *C. jejuni*; eugenol and trans-cinnamaldehyde mixture: chicks fed a basal diet supplemented with a mixture of eugenol and trans-cinnamaldehyde; and cefoxitin: chicks fed a basal diet and treated with cefoxitin antibiotic. All the groups, except the NC group, were challenged with XDR *C. jejuni* at 23 days of age. ^a–c^ Means of the columns with different letters indicate a statistically significant difference (*p* < 0.05).

The expression levels of the *C. jejuni flaA*, *virB11*, and *wlaN* virulence genes after treatment with the mixture of eugenol and trans-cinnamaldehyde and cefoxitin at 7 and 14 dpi are shown in Figure 6. The *flaA*, *virB11*, and *wlaN* mRNA expression levels were significantly (*p* < 0.05) downregulated in the cefoxitin, followed by the mixture of eugenol and trans-cinnamaldehyde-treated groups, compared to the PC group at both time points. Interestingly, no statistical significance variations were observed among the expression levels of *C. jejuni flaA, virB11*, the and *wlaN* genes between the cefoxitin- and mixture of eugenol and trans-cinnamaldehyde-treated groups at 14 dpi. Additionally, the *flaA* relative expression level was significantly (*p* < 0.05) decreased in the chicks treated with cefoxitin and the mixture of eugenol and trans-cinnamaldehyde at 7 dpi, with respect to the PC group, with no statistically significant differences between the latter groups.

#### 3.5.4. Quantification of the *C. jejuni* DNA Copies

**Figure 7:**
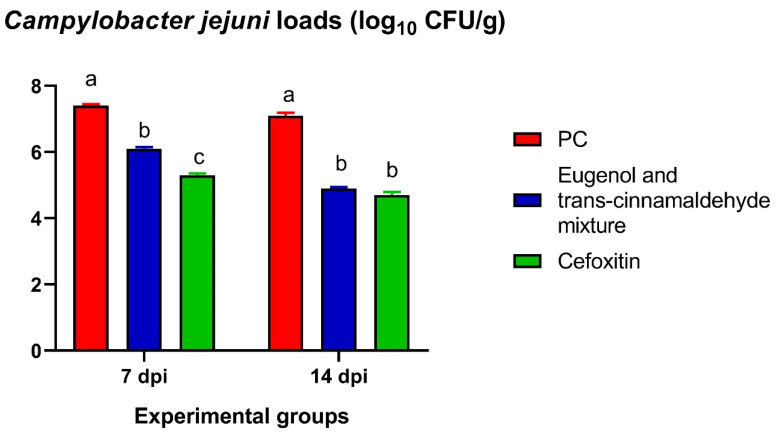
Quantification of the fecal *Campylobacter jejuni* loads (log_10_ CFU) in response to supplementing the mixture of eugenol and trans-cinnamaldehyde and treatment with cefoxitin antibiotic at 7 days (7 dpi) and 14 days post-infection (14 dpi) with XDR *C. jejuni*, as were determined via the qPCR assay. The results are expressed as the means ± standard error of the mean (SEM, error bars). PC (positive control): chicks fed a basal diet and challenged with XDR *C. jejuni*; eugenol and trans-cinnamaldehyde mixture: chicks fed a basal diet supplemented with a mixture of eugenol and trans-cinnamaldehyde; and cefoxitin: chicks fed a basal diet supplemented with a cefoxitin antibiotic. All groups, except the NC group, were challenged with XDR *C. jejuni* at 23 days of age. ^a–c^ Means of the columns with different letters indicate a statistically significant difference (*p* < 0.05).

The quantification data of *C. jejuni* in the cecal contents of chicks are presented in Figure 7. The most significant (*p* < 0.05) reduction in *C. jejuni* loads was detected in the chicks treated with cefoxitin, followed by the mixture of eugenol and trans-cinnamaldehyde, compared to the PC group at 7 days post-challenge with XDR *C. jejuni*. Interestingly, no statistical significance variations were observed among the log_10_ copies of the *C. jejuni* populations in the cecal contents of the chicks treated with the mixture of eugenol and trans-cinnamaldehyde and cefoxitin at 14 dpi.

## 4. Discussion

It was stated that multiple worldwide crises were established as a result of the extensive spread of antimicrobial-resistant bacteria, including methicillin-resistant *Staphylococcus aureus* (MRSA), vancomycin-resistant *Staphylococcus aureus* (VRSA), *Klebsiella* spp., *Mycoplasma* spp., as well as zoonotic foodborne bacteria, such as *Campylobacter* spp., *Salmonella* Enteritidis, *Salmonella* Typhimurium, and *E. coli* [40,41,42,43,44,45,46,47,48]. *Campylobacter* spp., as commensal bacteria in the intestinal tract of domestic animals and chickens, are recognized as the primary sources of campylobacter infections in humans. Recently, *Campylobacter* spp. have become resistant to several antimicrobials, especially macrolides, FQ, and tetracyclines being important public health problems around the world. In the current work, we determined the high incidence of campylobacter isolates (67.3%) between different chicken specimens in Sharkia Governorate, Egypt. Our results are higher than those observed in previous studies carried out in Egypt, (7.6%) [49], (20.3%) [50], (26.9%) [51], and (32.8%) [7], meanwhile, these findings are consistent with the results of a recent study carried out in Kenya (69%) [52]. In the current work, *Campylobacter* spp. were more prevalent between the cloacal swabs (90%). These results are higher than those observed in India (71%) [53], Kenya (69%) [52], and Egypt (54.3%) [7]. In the present study, *Campylobacter* spp. were not detected in the chicken luncheon meats (0%), which is similar to the results of a previous study conducted in Egypt [54]. Additionally, *C. jejuni* was the most commonly recognized spp. (54%), which is consistent with the findings of previous studies carried out in Tunisia (68.9%) [55], and China; (20.3%) [56] and (12.3%) [12], respectively. Generally, the differences in the prevalence of *Campylobacter* spp. among the different studies might be correlated to the contamination and health conditions, climate factors, the geographical locations, the sources of tested samples, and the conventional identification methods [57].

Unsurprisingly, there are differences in the antibiotic resistance profiles between different countries, as a result of the variations in the prescribed antibiotics. Of note, our examined isolates showed complete resistance to ampicillin and erythromycin (100% each). These results are higher than those observed in other studies conducted in South Korea (57.4 and 14.9%, respectively) [58] and Pakistan (15 and 10%, respectively) [59]. Herein, high resistance levels to doxycycline and ciprofloxacin were observed among our examined isolates (83.2 and 79.2%, respectively); these results were lower than those reported in a previous study conducted in Tunisia (100 and 99.2%, respectively) [55]. Alarmingly, all global warnings are related to the emergence of MDR strains, but, recently, the XDR spread is taking concrete steps. In the current study, 74.3 and 25.7% of the examined campylobacter isolates were identified as MDR and XDR, respectively. These results are consistent with those detected in a recent study carried out in Egypt, where 28.5 and 69% of the tested isolates were MDR and XDR [54]. Herein, our examined isolates had MAR indices ≥ 0.39. These results were higher than those observed in another study conducted in South Korea, where all the tested isolates had MAR indices ≤ 0.2 [58]. In developing countries, the high resistance rates of campylobacters could result from the excessive uncontrolled utilization of antimicrobial agents in animal and human treatments without any prescription and as growth promoters in veterinary medicine. Moreover, our findings show alarmingly high resistance prevalence of doxycycline, erythromycin, and ciprofloxacin because they are the antibiotics of choice for the treatment of human campylobacter infection resulting in significant problems, where antimicrobial treatment becomes limited. Thus, the utilization of antimicrobials should be controlled in humans and animals [4]. Thus, it is significant to utilize medicinal plants, such as phytochemicals, as an alternative to the antimicrobials [60,61,62].

Out of the 26 molecularly investigated campylobacter isolates, 19 (73.1%) and 7 (26.9%) were positive for *mapA* and *ceuE* genes, respectively, being identified as *C. jejuni* and *C. coli*, respectively. Interestingly, there was a 100% correlation between the molecular and conventional identification results. These results were in agreement with a previous study conducted in Egypt, where 89.5% of the tested isolates were identified as *C. jejuni*, while the remaining isolates (10.5%) were identified as *C. coli* [63].

Herein, 50% of the examined campylobacter isolates were positive for the *virB11* gene. These results are in contrast with the results of another study conducted in South Korea, where all the examined isolates were negative to the *virB11* gene [64]. These differences in the prevalence of the *virB11* gene may be attributed to the isolates’ sources and samples’ types.

Interestingly, our results reveal that all the examined campylobacter isolates (100%) are positive for the *flaA* gene. These findings are in complete agreement with a previous study conducted in Egypt [49], suggesting that the *flaA* gene has a significant virulence role in campylobacter isolates due to its significant part in the invasion, adhesion, and motility *Campylobacter* spp.

In the current work, the dietary supplementation of a mixture of eugenol and trans-cinnamaldehyde was able to modulate the immune responses of broiler chickens to overcome experimental *C. jejuni* infection and enhance their growth performance. In the present study, the mixture of eugenol and trans-cinnamaldehyde enhanced the overall growth rate and restored the decreased body weight and impaired the FCR post-infection with XDR *C. jejuni*, in comparison to the PC group. In agreement with our findings, a recent study reported the growth-promoting effect of carvacrol in broiler chickens challenged with *C. jejuni* [65]; however, the efficacy of the mixture of eugenol and trans-cinnamaldehyde on the broiler chickens’ performance after the experimental infection with the XDR *C. jejuni* strain has not been studied until now. Additionally, another study reported that broiler chicks fed with various levels of EOs exhibited an improved FCR and BWG after experimental infection, when compared to the PC group [66]. Moreover, the dietary supplementation of plant-active principles had better growth performance variables and *C. jejuni* load in both intestinal content and excreta [67]. According to previous studies, the positive effects of EOs on broiler chickens’ growth performance might result from the capability of their bioactive compounds to enhance their immune functions [68], stimulate their appetite and antimicrobial characteristics [69]. However, the EO extracts’ mode of actions are not yet defined and could vary as a result of the different forms, sources, and structures of the active components being utilized in diets [66].

Cytokines have significant regulatory roles in the inflammatory response of the intestinal tract. When the pathogens invade the gastrointestinal epithelial cells, the immune cells of the intestinal tract are triggered to produce cytokines that have fundamental roles in the host immune responses against microorganisms [70]. The *TNF-α* is a significant proinflammatory cytokine that regulates the host immune response against microorganisms via differentiating and proliferating the immune cells [71], but the excessive and prolonged secretion of proinflammatory cytokines might result in gastrointestinal damage [72]. Moreover, *TNF-α*, *IL-2, IL-6*, and *IL-8* trigger an inflammatory response through recruiting the antimicrobial cells, including macrophages and neutrophils [71]. In the current study, the splenic proinflammatory cytokine (*IL-2*, *IL-6*, *IL-8*, and *TNF-α*) gene expression levels were upregulated in chicks that received a control basal diet and challenged experimentally with *C. jejuni*; however, the relative expression levels of *IL-6* and *IL-8* genes were significantly decreased in the cefoxitin-treated group, followed by the mixture of the eugenol and trans-cinnamaldehyde-supplemented group concerning the PC group. Additionally, supplementing our challenged birds with a mixture of eugenol and trans-cinnamaldehyde had regulatory effects on the proinflammatory cytokine genes expressions through a significant downregulation of *TNF-α* and *IL-2* expression levels, which may counteract the inflammatory effects caused by *C. jejuni*, indicating their immunostimulant activities. This may result from the role of dietary EOs in improving the host non-specific immune response via the nonspecific killing of parasites, tumor cells, bacteria, and fungi, which lead to minimizing the microorganism loads [73]. Meanwhile, *IL-10* has mainly opposed and complex effects on the immune and inflammatory responses via suppressing their actions [74]. In the current study, the highest expression level of the *IL-10* gene was observed in the mixture of the eugenol and trans-cinnamaldehyde-supplemented group, which may minimize the excessive inflammatory response indicating their significant anti-inflammatory characteristics. In agreement with our results, recent studies showed the immunostimulant and anti-inflammatory effects of Eos via minimizing the expression levels of *TNF-α* [19], *IL-2*, and *IL-6* [66] genes and decreasing that of the *IL-10* gene [66] in broiler chicks challenged with *Salmonella* spp. due to their regulatory effects; however, the anti-inflammatory and immunostimulant effects of the mixture of eugenol and trans-cinnamaldehyde in broiler chickens challenged with the XDR *C. jejuni* strain has not yet been investigated.

Targeting the virulence factors of microorganisms through utilizing novel anti-virulence treatments is a promising alternative method, which could be utilized to disarm the bacterial microorganisms [60]. Following the previous statement, our data show that the mixture of eugenol and trans-cinnamaldehyde downregulated the expression levels of *C. jejuni flaA*, *wlaN* and *virB11* virulence genes in challenged broilers concerning the PC group. In agreement with our results, recent studies reported the in vivo anti-virulence effects of EOs via suppressing the virulence genes’ expressions of *Salmonella* spp. in challenged broiler chicks [19]. Previous studies were continuously reporting the modulatory effects of EOs on *C. jejuni* virulence genes’ expressions in vitro [7,17,18]; however, to the best of our knowledge, there are no studies reporting the in vivo efficacy of the mixture of eugenol and trans-cinnamaldehyde on *C. jejuni* virulence in challenged broiler chickens.

*Campylobacter* spp. are commensal bacteria in the gastrointestinal tract of poultry and chickens, which considered the main reservoir for human campylobacter infections [1]. Herein, the quantitative analysis of cecal *C. jejuni* in challenged broilers showed that the dietary supplementation of a mixture of eugenol and trans-cinnamaldehyde significantly (*p* < 0.05) minimized *C. jejuni* populations 7 and 14 dpi, in comparison to the PC group. Our findings are in agreement with the results of previous studies, which demonstrate that the dietary supplementation of carvacrol significantly (*p* < 0.05) decreases *C. jejuni* counts in ceca [65], cloacal swabs, and the colon [75] of challenged broiler chickens, in comparison to the PC group. Additionally, a previous study reported a reduction in the cecal *C. jejuni* counts in challenged broilers after dietary supplementation with an EO mixture containing eugenol, indicating their inhibitory effects against *C. jejuni* [76]; however, the efficacy of the mixture of eugenol and trans-cinnamaldehyde on cecal *C. jejuni* loads in broiler chickens challenged with the XDR *C. jejuni* strain has not been explored to date.

## 5. Conclusions

The current work shows an alarming prevalence of avian XDR *Campylobacter* spp. in Egypt. The *wlaN* and *virB11* genes were more prevalent among the examined campylobacter isolates from chicken cecal parts and cloacal swabs. Interestingly, our findings clearly prove the beneficial effects of a mixture of eugenol and trans-cinnamaldehyde on broiler chickens’ growth performance through decreasing the fecal load of *C. jejuni* and modulating the expression of proinflammatory and anti-inflammatory cytokines and decreasing the excessive inflammatory response. Furthermore, the administration of the mixture of eugenol and trans-cinnamaldehyde for broilers challenged with XDR *C. jejuni* had immunostimulant, anti-inflammatory, antimicrobial and anti-virulence effects. Therefore, our findings recommend the application of the mixture of eugenol and trans-cinnamaldehyde as an alternative to antimicrobials for the treatment and control of *Campylobacter* spp. infection in the veterinary fields, as well as for immunocompromised individuals.

## Figures and Tables

**Table 1:** Sequences of the oligonucleotide primers targeting six genes of the *Campylobacter* species and their respective amplified PCR products.

Specificity (Target Gene)	Primer Sequence (5’-3’)	Amplified Product (bp)	Denaturation Temperature	References
Genus *Campylobacter* (*23S rRNA*)	F: TATACCGGTAAGGAGTGCTGGAG	650	95 °C	[28,32]
	R: ATCAATTAACCTTCGAGCACCG			
*Campylobacter jejuni* (*mapA*)	F: CTATTTTATTTTTGAGTGCTTGTG	589	95 °C	[29,33]
	R: GCTTTATTTGCCATTTGTTTTATTA			
*Campylobacter coli* (*ceuE*)	F: AATTGA AAATTG CTCCAACTATG	462	95 °C	[29,33]
	R: TGATTT TATTATTTGTAGCAGCG			
FlaA (*fla*A)	F: AATAAAAATGCTGATAAAACAGGTG	855	94 °C	[30,34]
	R: TACCGAACCAATGTCTGCTCTGATT			
VirB (*vir*B11)	F: TCTTGTGAGTTGCCTTACCCCTTTT	494	94 °C	[30,34]
	R: CCTGCGTGTCCTGTGTTATTTACCC			
WlaN (*wla*N)	F: TTAAGAGCAAGATATGAAGGTG	672	94 °C	[32,35]
	R: CCATTTGAATTGATATTTTTG			

**Table 2:** Ingredients and nutrient composition of the basal diet.

Ingredient (%)	Starter (Days 1–10)	Grower (Days 11–20)	Finisher (Days 21–37)
Yellow corn	60.10	64.00	65.70
Soybean oil	1.57	2.50	4.20
Soybean meal	34.5	29.3	25.90
Common salt	0.3	0.3	0.3
Calcium diphasic phosphate	1.2	1.2	1.2
Calcium carbonate	1.1	1.1	1.1
Premix *	0.8	0.8	0.8
Choline chloride	0.2	0.2	0.2
DL-methionine 99%	0.17	0.17	0.17
L-lysine HCL 78%	0.33	0.33	0.33
Anti-mycotoxin	0.1	0.1	0.1
Calculated composition			
ME (kcal/kg)	3001	3100	3222
CF (%)	2.64	2.50	2.46
EE (%)	4.07	5.14	6.83
CP (%)	23.01	21.02	19.57
Available P (%)	0.49	0.44	0.42
Ca (%)	1.02	1.04	1.03
Methionine (%)	0.500	0.44	0.46
Lysine (%)	1.41	1.28	1.18

* Vitamin premix supplied per kilogram of diet: Mn (sulphate and oxide), 100 mg; Zn (sulphate and oxide), 120 mg; I (iodide), 1.2 mg; Se (selenate), 0.28 mg; Cu (sulphate), 14 mg; Fe (sulphate), 30 mg; cyanocobalamin, 15 μg; biotin, 300 μg; pyridoxine, 6 mg; folate, 3 mg; niacin, 50 mg; pantothenate, 12 mg; thiamine, 4 mg; riboflavin, 7 mg; menadione, 3.5 mg; cholecalciferol, 6500 IU; tocopherol acetate, 75 mg; retinol, 12.000 IU; ME: metabolizable energy; CF: crude fiber; EE: ether extract; CP: crude protein; P: phosphorus; and Ca: calcium.

**Table 3:** Primer sequences of the cytokine-related genes used in the reverse transcription quantitative real-time PCR assay.

Target Gene	Primer Sequence (5’-3’)	Accession No.
*TNF-α*	F: CTGCACTTCAGGGTGATCG	XM_008262537.2
	R: CTACGTGGGCTAGAGGCTTG	
*IL-2*	F: GCTTATGGAGCATCTCTATCATCA	XM_015276098.2
	R: GGTGCACTCCTGGGTCTC	
*IL-6*	F: GCCAACCCTACAACAAGA	NC_013678
	R: AGAGCCACAACGACTGAC	
*IL-8*	F: CTCTCTTGGCAACCTTCCTG	KT216053.1
	R: TTGCACAGTGAGGTCCACTC	
*IL-10*	F: AAAAGCTAAAAGCCCCAGGA	NM001082045.1
	R: CGGGAGCTGAGGTATCAGAG	
*GAPDH*	F: TGTTTGTGATGGGCGTGAA	NC_013676.1
	R: CCTCCACAATGCCGAAGT	

*TNF-α:* tumor necrosis factor-alpha; *IL*: interleukin; and *GAPDH*: glyceraldehyde 3-phosphate dehydrogenase.

**Table 4:** Prevalence of *Campylobacter* species in various chicken samples in Sharkia Governorate, Egypt.

Sample Type (Symbol, No.)	Total No. of Campylobacter Isolates (%) *	No. of *Campylobacter* spp. (%) *	
		*C. jejuni*	*C. coli*
Chicken luncheon meats (Clm, 30)	0	0	0
Chicken livers (Cl, 30)	25 (83.3)	19 (63.3)	6 (20)
Chicken breast meats (Cbm, 30)	23 (76.7)	20 (66.7)	3 (10)
Chicken cecal parts (Ccp, 30)	26 (86.7)	21 (70)	5 (16.7)
Chicken cloacal swabs (Ccs, 30)	27 (90)	21 (70)	6 (20)
Total (150)	101 (67.3)	81 (54)	20 (13.3)

* The isolation rates were calculated regarding the total number of the examined samples.

**Table 5:** Antimicrobial resistance patterns of the *Campylobacter* species from various chicken sources.

Antimicrobial Class	Antimicrobial Agent	No. of Resistant Campylobacter Isolates from Various Chicken Sources (%)				Total No. of Campylobacter Isolates (%) (*n* = 101)	*p*-Value
		Liver (*n* = 25)	Breast Meats (*n* = 23)	Cecal Parts (*n* = 26)	Cloacal Swabs (*n* = 27)		
Lincosamide	Clindamycin	24 (96)	20 (87)	25 (96.2)	27 (100)	96 (95)	0.18
Polypeptides	Colistin	19 (76)	17 (73.9)	22 (84.6)	17 (63)	75 (74.3)	0.348
Phenicols	Chloramphenicol	24 (96)	16 (69.6)	18 (69.2)	20 (74.1)	78 (77.2)	0.076
Oxazolidones	Linezolid	23 (92)	17 (73.9)	21 (80.8)	12 (44.4)	73 (72.3)	0.001 **
Aminoglycosides	Amikacin	9 (36)	9 (39.1)	7 (26.9)	10 (37)	35 (34.7)	0.821
	Gentamicin	21 (84)	21 (91.3)	16 (61.5)	21 (77.8)	79 (78.2)	0.07
	Tobramycin	19 (76)	15 (65.2)	20 (76.9)	16 (59.3)	70 (69.3)	0.458
Macrolides	Azithromycin	23 (92)	22 (95.7)	23 (88.5)	24 (88.9)	92 (91.1)	0.871
	Erythromycin	25 (100)	23 (100)	26 (100)	27 (100)	101 (100)	NA
Tetracyclines	Doxycycline	23 (92)	18 (78.3)	20 (76.9)	23 (85.2)	84 (83.2)	0.472
Sulphonamides	Trimethoprim-sulfamethoxazole	25 (100)	23 (100)	26 (100)	27 (100)	101 (100)	NA
Quinolones	Ciprofloxacin	20 (80)	19 (82.6)	21 (80.8)	20 (74.1)	80 (79.2)	0.903
	Nalidixic acid	25 (100)	23 (100)	23 (88.5)	25 (92.6)	96 (95)	0.04 *
Beta-lactams	Aztreonam	18 (72)	21 (91.3)	21 (80.8)	25 (92.6)	85 (84.2)	0.015 *
	Imipenem	14 (56)	11 (47.8)	7 (26.9)	11 (40.7)	43 (42.6)	0.195
	Cefoxitin	13 (52)	8 (34.8)	8 (30.8)	5 (18.5)	34 (33.7)	0.047 *
	Sulbactam-ampicillin	18 (72)	17 (73.9)	13 (50)	13 (48.1)	61 (60.4)	0.114
	Ampicillin	25 (100)	23 (100)	26 (100)	27 (100)	101 (100)	NA

*n*: number, * and ** represent the statistically significant differences in the resistance profiles among the campylobacter isolates from different chicken sources to the indicated antimicrobial agent using a chi-squared test; * *p* < 0.05, ** *p* < 0.01, NA: non-applicable.

**Table 6:** Multiple antibiotic resistance indices and the frequency of resistance of the campylobacter isolates from different chicken sources.

MAR Index	No. of Antimicrobials to Which the Isolates Were Resistant *	No. of AMC	No. of Resistant Campylobacter Isolates from Different Chicken Sources (%)				Total No. of Campylobacter Isolates (%) (*n* = 101)	The Character of Resistant Strains
			Liver (*n* = 25)	Breast Meats (*n* = 23)	Cecal Parts (*n* = 26)	Cloacal Swabs (*n* = 27)		
0.39	7	7	-	-	-	1 (3.7)	1 (1)	MDR
0.44	8	7	-	-	2 (7.7)	-	2 (2)	
0.56	10	6	-	-	-	3 (11.1)	3 (3)	
		7	1 (4)	2 (8.7)	1 (3.8)	1 (3.7)	5 (5)	
0.61	11	8	1 (4)	2 (8.7)	1 (3.8)	1 (3.7)	5 (5)	
		9	-	-	1 (3.8)	1 (3.7)	2 (2)	
0.67	12	7	-	1 (4.3)	1 (3.8)	-	2 (2)	
		8	-	-	1 (3.8)	2 (7.4)	3 (3)	
		9	-	-	2 (7.7)	-	2 (2)	
0.72	13	8	-	-	-	2 (7.4)	2 (2)	
		9	2 (8)	2 (8.7)	3 (11.5)	2 (7.4)	9 (8.9)	
		10	2 (8)	-	-	2 (7.4)	4 (4)	
0.78	14	8	-	-	1 (3.8)	-	1 (1)	
		9	1 (4)	-	3 (11.5)	2 (7.4)	6 (5.9)	
		10	2 (8)	4 (17.4)	2 (7.7)	1 (3.7)	9 (8.9)	
0.83	15	8	-	1 (4.3)	-	2 (7.4)	3 (3)	
		9	2 (8)	4 (17.4)	2 (7.7)	4 (14.8)	12 (11.9)	
		10	2 (8)	1 (4.3)	1 (3.8)	-	4 (4)	
0.89	16	8	-	3 (13)	-	-	3 (3)	XDR
		9	3 (12)	-	-	1 (3.7)	4 (4)	
		10	7 (28)	-	4 (15.4)	-	11 (10.9)	
0.94	17	10	2 (8)	3 (13)	1 (3.8)	2 (7.4)	8 (7.9)	

MAR: multiple antibiotic resistance; AMC: antimicrobial classes; *n*: number; MDR: multi-drug resistant; XDR: extensively drug resistant; * all the tested isolates were resistant to at least 7 antimicrobials.

**Table 7:** Effects of supplementing the mixture of eugenol and trans-cinnamaldehyde and cefoxitin treatment on the growth performance variables of the *C. jejuni*-challenged broilers over a 37-day experimental period.

	Experimental Group				*p*-Value	SEM
	NC	PC	Eugenol and Trans-Cinnamaldehyde Mixture	Cefoxitin		
BW, g/bird	2372 ^a^	1873 ^c^	2355 ^ab^	2363 ^a^	0.03	5.44
BWG, g/bird	2327 ^a^	1830 ^c^	2311 ^ab^	2319 ^a^	0.04	6.74
FI, g/bird	3843 ^b^	3998 ^a^	3884 ^b^	4075 ^a^	0.001	7.60
FCR	1.65 ^c^	2.18 ^a^	1.68 ^bc^	1.76 ^b^	0.001	0.05

BW: body weight; BWG: body weight gain; FI: feed intake; FCR: feed conversion ratio; PC (positive control): chicks fed a basal diet and challenged with *C. jejuni* at 23 days of age; NC (negative control): chicks fed a basal diet; and SEM: standard error of the mean. Means with no common superscripts within the same row differ significantly (*p* < 0.05) using Tukey’s test and the a, ab, and b indexes from Tukey’s test as corresponding values.

## Data Availability

The data presented in this study are available on request from the corresponding author.
